# Amh/Amhr2 Signaling Causes Masculinization by Inhibiting Estrogen Synthesis during Gonadal Sex Differentiation in Japanese Flounder (*Paralichthys olivaceus*)

**DOI:** 10.3390/ijms24032480

**Published:** 2023-01-27

**Authors:** Toshiya Yamaguchi, Takeshi Kitano

**Affiliations:** 1 Nansei Field Station, National Research and Development Agency, Japan Fisheries Research and Education Agency, Minamiise, Mie 516-0193, Japan; 2 Department of Biological Sciences, Graduate School of Science and Technology, Kumamoto University, Kumamoto 860-8555, Japan

**Keywords:** Japanese flounder, *amh*, *amhr2*, estrogen, gonad, sex differentiation, CRISPR/Cas9

## Abstract

The anti-Müllerian hormone (Amh) is a protein belonging to the TGF-β superfamily, the function of which has been considered important for male sex differentiation in vertebrates. The Japanese flounder (*Paralichthys olivaceus*) is a teleost fish that has an XX/XY sex determination system and temperature-dependent sex determination. In this species, *amh* expression is up-regulated in genetic males and in temperature-induced masculinization during the sex differentiation period. However, to the best of our knowledge, no reports on the Amh receptor (Amhr2) in flounder have been published, and the details of Amh signaling remain unclear. In this study, we produced *amhr2*-deficient mutants using the CRISPR/Cas9 system and analyzed the gonadal phenotypes and sex-related genes. The results revealed that the gonads of genetically male *amhr2* mutants featured typical ovaries, and the sex differentiation-related genes showed a female expression pattern. Thus, the loss of Amhr2 function causes male-to-female sex reversal in Japanese flounder. Moreover, the treatment of genetically male *amhr2* mutants with an aromatase inhibitor fadrozole, which inhibits estrogen synthesis, resulted in testicular formation. These results strongly suggest that Amh/Amhr2 signaling causes masculinization by inhibiting estrogen synthesis during gonadal sex differentiation in the flounder.

## 1. Introduction

Most teleosts exhibit gonochorism, along with the presence of regular males and females such as other vertebrates. Meanwhile, some teleosts are known to have diverse reproductive systems because sex is determined not only by genetic factors, but also by environmental factors including temperature, pH, density, and oxygen concentration [1]. These factors act as master switches that can direct bipotential gonadal primordia to become ovary or testis by activating differentiation pathways. Thus, teleost sex determination can involve genetic sex determination (GSD), environmental sex determination (ESD), or a combination of both of them.

The Japanese flounder (*Paralichthys olivaceus*), a marine culture fish that is economically important for aquaculture in Asia, is a teleost fish that has an XX/XY sex determination system [2]. However, XX flounder can be completely sex-reversed to become phenotypic males by rearing the larvae at high or low water temperatures early in the sex differentiation period at 50–60 days after hatching (dah) [2,3,4]. Therefore, the Japanese flounder exhibits both GSD and temperature-dependent sex determination (TSD) as a kind of ESD. This makes it an excellent model to study the molecular mechanisms determining the relationship between GSD and TSD [5,6]. In our previous study on TSD, the treatment with an aromatase inhibitor (fadrozole) or an anti-estrogen agent (tamoxifen) and a high temperature induced the masculinization of XX flounder and suppressed the mRNA expression of ovarian aromatase (*cyp19a1*), a steroidogenic enzyme that converts androgen into estrogen [7,8]. Furthermore, estrogen (17β-estradiol) administration completely inhibited the masculinization due to a high temperature [9]. It is suggested that the suppression of *cyp19a1* mRNA expression and the inhibition of estrogen biosynthesis may ultimately trigger the gonadal masculinization of XX flounder induced by a high temperature.

The transcript of the *cyp19a1* gene is reported to be expressed in a female-specific manner during sexual differentiation in various teleosts including flounder [6,8]. We previously investigated the molecular mechanism of the regulation of *cyp19a1* expression, and we reported that the transcription factor forkhead box protein l2 (Foxl2) and follicle-stimulating hormone (FSH) signal may positively regulate the expression of *cyp19a1* [10,11]. The *foxl2* mRNA is specifically expressed in females in the gonad during the sex differentiation period, which is similar to *cyp19a1*, and in vitro experiments have shown that Foxl2 protein directly binds to the transcriptional regulatory promoter present in the 5’ upstream region of the *cyp19a1* gene, and it positively regulates the promoter region. FSH is secreted from the pituitary gland, and signals through receptors (Fshr) present in gonadal somatic cells [12,13]. In other teleosts, the transcription factor *foxl2* is also expressed in a female-specific manner in the gonads [14]. Thus, it is thought that Foxl2 and FSH signals are present upstream of the *cyp19a1* gene regulatory cascade and induce feminization in flounder during sexual differentiation. Meanwhile, we previously reported that cortisol, a stress response hormone, is involved in the molecular mechanism of gonad masculinization during treatments with high temperatures [11]. It was shown that cortisol, the secretion of which increases in response to stress, inhibited the transcriptional regulation of the *cyp19a1* gene via the FSH signal in Japanese flounder. Cortisol has also been reported to induce masculinization in medaka (*Oryzias latipes*) [15] and pejerrey (*Odontesthes hatcheri*) [16]. Thus, the mechanism of TSD is explained by the expression of the estrogen biosynthetic *cyp19a1* gene and the antagonism of this by the stress response hormone cortisol. However, the mechanism by which differentiation into male gonads occurs in flounder with the XX/XY sex determination system has not been elucidated.

In Japanese flounder, the anti-Müllerian hormone (*amh*) mRNA is reported to be expressed in a male-specific manner, which is in contrast to *cyp19a1*, during sexual differentiation [17]. Amh, which is also known as Müllerian inhibiting substance (Mis), is a protein belonging to the TGF-β superfamily [18]. In mammals, a distinctive function of Amh is the induction of Müllerian duct regression during early ovarian differentiation [19,20]. The expression of Amh is restricted to the somatic cells in contact with germ cells, namely, Sertoli cells in the testis and granulosa cells in the ovary [21,22]. Amh primarily functions through the anti-Müllerian hormone receptor type 2 (Amhr2) and via a single-transmembrane domain and a serine/threonine kinase domain [23]. The expression of *amhr2* is restricted to Sertoli cells in the testis and granulosa cells in the ovary.

Recently, Y-chromosome allele-specific *amh* (*amhy*) has been reported to be the master sex-determining gene in flounder [24]. A genomic analysis based on sex-specific SNP information revealed the presence of a Y-allele-specific deletion in the promoter region of the *amh* gene. A genotype-sex association analysis found a identify the association between phenotypic sex and genotype. Moreover, experiments involving the loss of function of *amh* produced using CRISPR/Cas9 resulted in the complete sex reversal of XY flounder, thus supporting the role of *amhy* as a sex-determining gene in flounder. The finding that *amhy* is the master sex-determining gene has enabled major progress in research on the molecular mechanisms of genetic sex determination in flounder. It has also become possible to determine whether the genetic sex is XX or XY without performing a mating test on Japanese flounder.

In this study, to investigate the molecular cascade of male gonadal differentiation in Japanese flounder, we generated *amhr2* mutant flounder using the CRISPR/Cas9 system [25]. The system induced the biallelic mutation of the target gene, resulting in phenotypic knockout in the F_0_ generation by inducing the wide deletion using two CRISPR RNAs (crRNAs) for the target gene. We also performed a sex-reversal experiment using the aromatase inhibitor fadrozole to confirm the function of estrogen during gonadal differentiation in *amh* mutants. These experiments showed a functional role of the *amhr2* gene in the genetic sex differentiation in Japanese flounder.

## 2. Results

### 2.1. Expression Analysis of amh and amhr2 in Gonads in XY and XX Flounder

**Figure 1:**
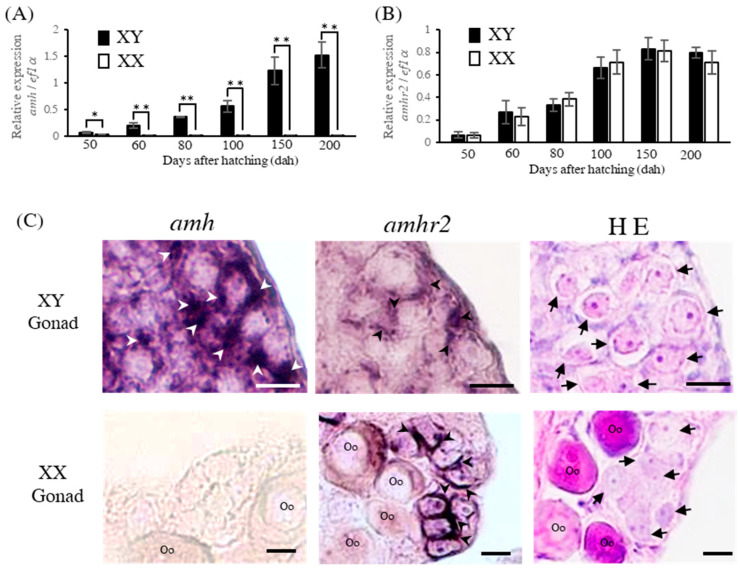
Expression analysis of *amh* and *amhr2* in XY and XX flounder. Real-time PCR analysis of *amh* (**A**) and *amhr2* (**B**) expression in XY and XX flounder from the sexually indifferent stage (50 dah) to the stage of clear differentiation in testis or ovary (200 dah). * *p* < 0.05, ** *p* < 0.01. (**C**) Cellular localizations of *amh* and *amhr2* in the flounder gonads (200 dah) by in situ hybridization. Arrowheads indicate signals positive for *amh* or *amhr2* transcripts, while arrows indicate gonium cells. Oo: oocyte. Scale bar: 20 μm.

We investigated the expression levels of amh and amhr2 transcripts using a quantitative real-time PCR from the sexually indifferent stage (50 dah) to the stage of clear differentiation in the testis or ovary (200 dah) and compared such expression levels between XY and XX flounder. The results showed that amh transcripts were detected in XY fish from 50 to 200 dah, whereas the level of transcripts in genetic females was very low throughout the whole period (Figure 1A). Meanwhile, the amhr2 transcript was detected in both of the genotypes from 50 to 200 dah, and the expression levels showed no difference between XY and XX fish during the same period (Figure 1B). The cellular localizations of amh and amhr2 transcripts were also investigated using in situ hybridization in the XY testis and XX ovary at 200 dah. The gonium cells with characteristic swollen round nuclei were found in both of the genotypes by using hematoxylin-eosin staining (Figure 1C). The localization of the amh transcripts showed positive signals in the testis but not in the ovary; the signals were observed in the presumptive Sertoli cells in contact with the gonium cells in the testis (Figure 1C). The positive signals of amhr2 were detected in both the testis and the ovary (Figure 1C). These signals were localized in presumptive Sertoli cells in testis as well as the amh transcript signals. In the ovary, the expression was detected in the somatic cells in cysts of the gonium cells. Thus, both amh and amhr2 were expressed in the Sertoli cells of XY gonads. 

### 2.2. Generation of amhr2-Deficient Mutant Using CRISPR/Cas9 System in Japanese Flounder

**Figure 2:**
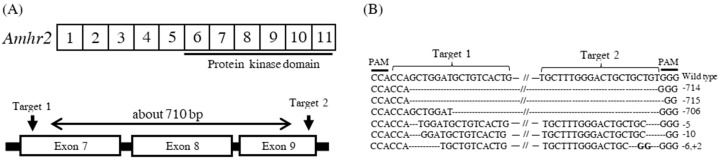
Construction of *amhr2*-mutant Japanese flounder using the CRISPR/Cas9 system. Schematic of positions targeted by crRNAs on the *amhr2* locus, showing exons (white boxes), introns (horizontal lines), and target positions (arrows) of the crRNAs with the CRISPR/Cas9 system (**A**). The target sequences of wild-type and *amhr2* mutants (**B**). The horizontal lines and dashed lines indicate wild-type sequences and deletions, respectively. Slashes indicate omission of long sequences. The numbers beside the target sequences indicate the approximate size of deletion (−) or insertion (+).

To investigate the function of the amhr2 gene, we produced amhr2-deficient mutant using the CRISPR/Cas9 system. This system can achieve the highly efficient production of the biallelic knockout mutant in the F_0_ generation by inducing a wide deletion using two crRNAs for the target gene as described previously [25]. The crRNAs were designed in exons seven and nine of the amhr2 gene, containing a conserved serine/threonine kinase domain. This was expected to produce a deletion of approximately 710 bp in the amhr2 gene, disrupting the function of the Amhr2 protein that it encodes (Figure 2A). The two crRNAs were co-injected with tracrRNA and Cas9 proteins into the fertilized eggs, and the mutants were reared to 200 dah to investigate the mutant genotype. To determine the detailed mutant genotype, we carried out a sequencing analysis for the amhr2 mutants using the DNA extracted from the gonad. From the results, representative amplicon fragment sequences showed deletions of 714, 715, or 706 bp between the two crRNA target sequences and a few base mutations in both of the target sequences (Figure 2B). The rates of this wide deletion among the sequenced fragments were 15/16, 13/16, and 15/16 clones in the genetic males and 8/8 and 6/8 clones in the genetic females (Table 1). Mutations were also identified in all of the clones analyzed by sequencing. Thus, wide deletions were induced in amhr2 mutants by using the CRISPR/Cas9 system in Japanese flounder.

### 2.3. amhr2 Mutants Showed Phenotypic Sex Reversal from Male to Female

**Figure 3:**
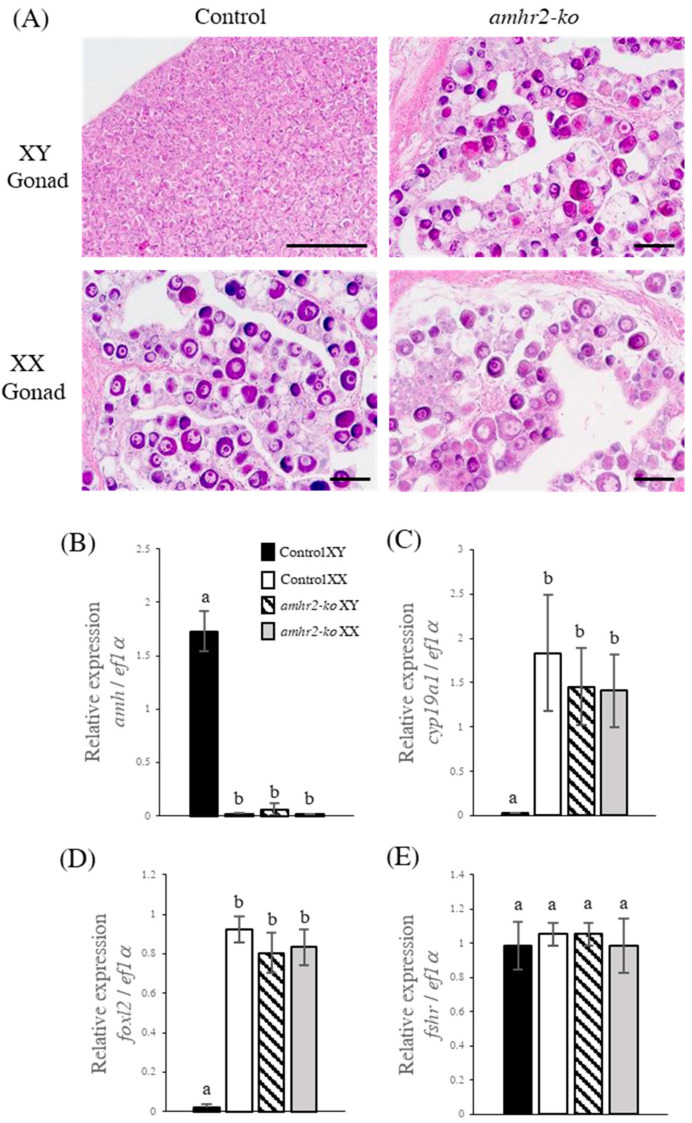
Phenotypic analysis of *amhr2* knockout mutants. Histological images of gonads from controls and *amhr2* mutants at 200 dah (**A**). Scale bar, 100 µm. Real-time PCR analyses of *amh* (**B**), *cyp19a1* (**C**), *foxl2* (**D**), and *fshr* (**E**) expressions were carried out using the gonads of the flounder at 200 dah. Different letters denote significant differences at *p* < 0.01.

We observed the phenotypes of the amhr2 mutants compared with those of the XY and XX flounder using a histological analysis. The wild-type XY gonad was assumed to consist of gonium cells from the morphological features of the cells, while the XX gonad showed an ovarian morphology, including several primary oocytes that were densely stained with eosin and an ovarian cavity (Figure 3A). Thus, the wild-type flounder gonad was differentiated into typical ovaries or testes at 200 dah. We then observed the phenotypes of the amhr2 mutants and compared them with the wild-type gonads. Both XY and XX amhr2 mutants showed primary oocytes and an ovarian cavity that are similar to those in the control group, and no histological differences were found between them (Figure 3A). All the XY amhr2 mutants thus showed a female phenotype with normal ovaries despite them being XY fish (Table 2). This showed that the suppression of the amhr2 gene function in the XY fish induced the phenotypic sex reversal from male to female. To examine the expression pattern of sex-related genes in the gonads of amhr2 mutants, a quantitative real-time PCR analysis was used to confirm the expressions of amh, cyp19a1, foxl2, and fshr in the XY and XX control and amhr2 mutants at 200 dah. amh showed significantly higher expression in the XY control fish than they did in the XX controls, whereas the expression levels were very low in the XY mutant fish, as they were in the XX mutants (Figure 3B). Meanwhile, the expression of cyp19a1 was very low in XY control fish, but it was significantly elevated in XY mutant fish compared with that in the XY controls (Figure 3C). The transcription factor foxl2, which up-regulates cyp19a1, was also expressed at a very low level in the XY control fish, whereas the expression was significantly elevated to be similar to that of cyp19a1 in the XY mutants (Figure 3D). There was no significant difference in fshr expression levels among all of the experimental groups (Figure 3E). Thus, the expression patterns of amh, cyp19a1, and foxl2 in the XX mutants were similar to those in the XX control fish. The expression patterns of the sex differentiation-related genes in the amhr2 mutants were confirmed to be feminized from the results of the histological phenotypic analysis. No significant differences in body length and body weight were observed between the amhr2 mutants and wild-type fish at 200 dah (Supplementary Table S1).

### 2.4. Effect of Fadrozole Treatment on Gonadal Phenotype of amhr2 Mutants

**Figure 4:**
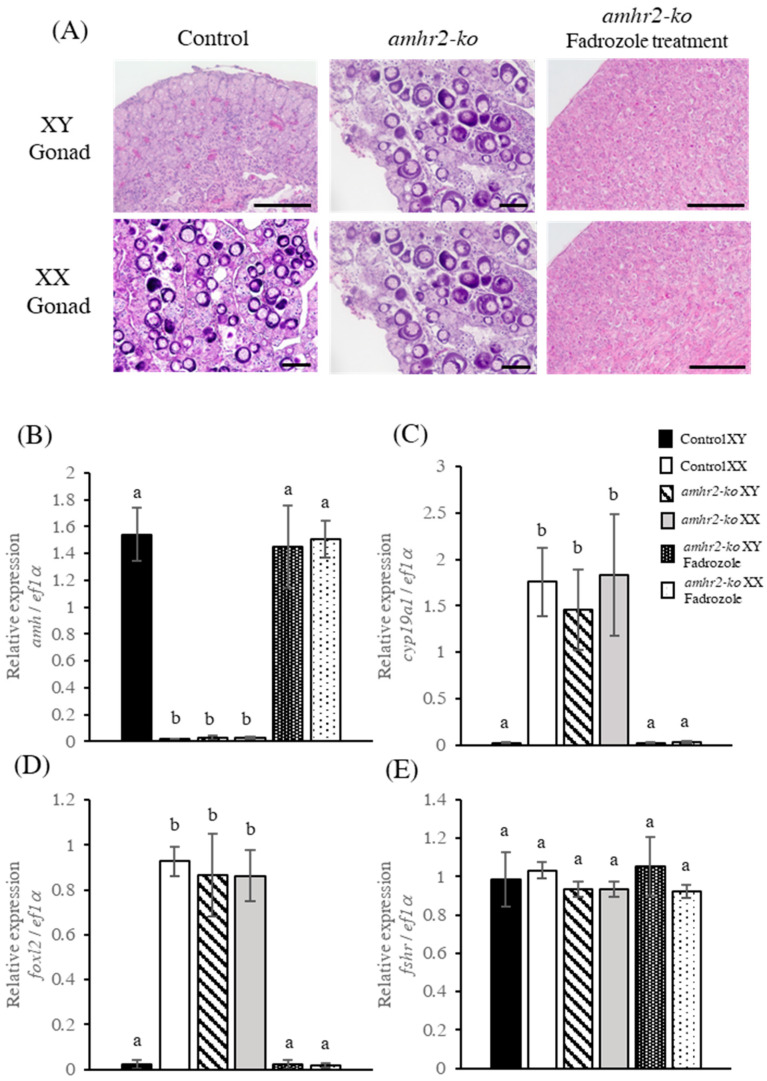
Phenotypic analysis of *amhr2* knockout mutants treated with the aromatase inhibitor fadrozole. Histological images of gonads from control fish, *amhr2* mutants, and fadrozole-treated *amhr2* mutants at 200 dah (**A**). Scale bar, 100 µm. Real-time PCR analyses of *amh* (**B**), *cyp19a1* (**C**), *foxl2* (**D**), and *fshr* (**E**) expressions were carried out using the gonads of the flounder at 200 dah. Different letters denote significant differences at *p* < 0.01.

The histological analysis revealed that the amhr2 mutants underwent sex reversal from male to female. To investigate the involvement of estrogens in the feminization of the amhr2 mutants, we administered fadrozole, which inhibits estrogen synthesis, to the amhr2 mutants. The larvae were treated with fadrozole for 30–100 dah, and a histological analysis was performed using the flounder at 200 dah. The results showed that the gonads of the XY amhr2 mutants exhibited normal ovaries, which were similar to those of the females in the control group, whereas the gonads of the fadrozole-treated XY amhr2 mutants exhibited normal testes (Figure 4A; Table 2). All of the fadrozole-treated XX amhr2 mutants also had typical testes in this experiment. Next, the expression of the sex differentiation-related genes was examined using the flounder at 200 dah by a real-time PCR. The results showed that amh was significantly up-regulated in the fadrozole-treated amhr2 mutants (Figure 4B), while cyp19a1 and foxl2 were prominently down-regulated (Figure 4C,D). Meanwhile, the fshr expression levels were not significantly different among all of the experimental groups (Figure 4E). These results indicate that estrogens are indispensable for the feminization of amhr2 mutants in Japanese flounder.

## 3. Discussion

In the present study, to elucidate the function of *amhr2* in the gonadal differentiation in Japanese flounder (*Paralichthys olivaceus*), we examined the expression pattern of its transcripts and confirmed transcript localization in the somatic cells in contact with gonium cells in both of the sexes. The loss of function of the *amhr2* gene produced using the CRISPR/Cas9 system led to a sex reversal from male to female in genetically male fish. The expression pattern of sex differentiation-related genes showed feminization that was similar to that of normal females. Furthermore, the treatment with fadrozole, which inhibits estrogen synthesis, caused the female-to-male sex reversal in the genetically male *amhr2* mutants.

Reports on *amh* in various teleost fish species have been published [17,26,27,28]. The function of this gene has been considered to be important for male sex differentiation in vertebrates. Based on quantitative real-time PCR, the *amh* expression in XY males was significantly higher than that in XX females from the sexually indifferent stage to gonadal post-differentiation stage (50–200 dah). These results were similar to the expression pattern of *amh* upon masculinization due to a high temperature in Japanese flounder [8,10]. Meanwhile, the expression levels of the *amhr2* transcripts showed no difference in the periods from sex differentiation to post-gonadal differentiation. Indeed, it was previously reported that *amhr2* does not exhibit sexually dimorphic expression during the gonadal differentiation period (50–80 dah) in Japanese flounder [24]. However, the expression of *amh* was shown to be higher in males than it was in females during sex differentiation in various teleost fish [17,26,27]. Based on in situ hybridization data, the *amh* and *amhr2* transcript signals were detected in the somatic cells in contact with gonium cells in Japanese flounder. In other vertebrates (e.g., chickens [29], medaka [30,31,32], and zebrafish [33]), *amh* and *amhr2* transcripts appeared in the Sertoli cells surrounding the male germ cells and in the follicle cells of female germ cells. Although we did not assess the protein expressions of Amh and Amhr2, it is speculated that *amh* and *amhr2* are also expressed in the somatic cells the surrounding germ cells and that Amh/Amhr2 signaling plays an important role in these cells during the sex differentiation period in Japanese flounder.

To investigate the functions of the *amhr2* gene in more detail, we produced an *amhr2* mutant using the CRISPR/Cas9 system. This system can achieve the highly efficient production of biallelic knockout mutants during F_0_ generation [25]. In the *amhr2* mutant flounder, large deletions were produced within the kinase domain of Amhr2, which is responsible for phosphorylation. Amhr2 is a type II receptor of the TGF-β superfamily, containing a single-transmembrane domain and an intercellular serine/threonine kinase domain [18]. Amh bound to Amhr2 was reported to induce the phosphorylation of type I receptors, transduce signals by phosphorylating Smad proteins, and regulate the transcription of downstream genes in mammals [34]. In medaka fish, a *hotei* mutant (*amhr2*-deficient) showed male-to-female sex reversal [35]. The mutant analysis identified a mutation in the *amhr2* gene, namely, an A-to-G mutation in exon 9, which is located in the receptor kinase domain. In the tiger pufferfish (*Takifugu rubripes*), a single-nucleotide polymorphism (SNP) in the *amhr2* gene was also reported to be associated with sex determination [36]. This SNP (C to G) in exon 9 changes an amino acid that is located in the receptor kinase domain from His to Asp. The findings showed that the kinase domain is critical for the function of Amhr2, suggesting that *amhr2* mutant flounder produced in this study shows a complete loss of function of Amh/Amhr2 signaling.

The Japanese flounder has an XX/XY sex determination system [2]. Recently, Y-chromosome allele-specific *amh* (*amhy*) was found to be the master sex-determining gene in flounder [24]. *amh*-mutant XY flounder showed phenotypic sex reversal from male to female, suggesting that Amh signaling is indispensable for the testicular differentiation in flounder. In mammals, the disruption of Amh signaling in Amh knockout XY mice led to partial hermaphroditism with premature follicle development [37]. Moreover, in Nile tilapia (*Oreochromis niloticus*), which also has an XX/XY sex determination system, there are two male-specific duplications of *amh*, which are designated *amhy* and *amhΔ-y* [38]. The knockout of *amhy*, *amhΔ-y*, and *amhr2* using the CRISPR/Cas9 system caused male-to-female sex reversal. Meanwhile, *amhr2*-deficient XY medaka showed sex reversal at a rate of more than 50% [35]. In this study, the loss of Amhr2 function causes male-to-female sex reversal in Japanese flounder. Thus, Amh/Amhr2 signaling may be responsible for male sex determination and testicular development in all vertebrates.

In vertebrates, estrogen plays an important role in ovarian differentiation and maintenance [13,39]. Foxl2 is a factor involved in female sex determination, which directly activates the expression of *cyp19a1* in vertebrates [10,40,41]. In medaka, *cyp19a1* expression is up-regulated in *amhr2*-deficient XY fish [35]. Similar results have reported that the knockdown of *amhy* in XY Patagonian pejerrey (*Odontesthes hatcheri*) resulted in the up-regulation of *foxl2* and *cyp19a1* expression [42]. Consistent with these reports, the knockout of *amhr2* in XY flounder resulted in increased *cyp19a1* and *foxl2* expression, along with normal ovary development. Therefore, to confirm the involvement of estrogens in *amhr2* mutants, we administered fadrozole, which inhibits estrogen synthesis. Fadrozole-treated XY *amhr2* mutants formed typical testes and exhibited the down-regulation of *cyp19a1* and *foxl2* transcripts. Previously, a fadrozole treatment caused complete female-to-male sex reversal with the down-regulation of *cyp19a1* expression in the flounder [7]. These results indicate that *amhr2*-deficient mutants display a phenotypic sex reversal from male to female by elevating the level of estrogens, along with increased *cyp19a1* and *foxl2* expression. Therefore, Amh/Amhr2 signaling appears to cause female-to-male sex reversal by inhibiting estrogen synthesis.

In Japanese flounder, female fish grow faster than the males do, and therefore, females are highly valued in the aquaculture industry [2,3]. In this study, although there was no significant difference in the body length and the body weight among all of the experimental groups at 200 dah (Supplementary Table S1), the sex difference in growth is likely to clarify after they reach 1 year of age [2]. Therefore, future studies will be needed to continuously research the growth of *amhr2* mutant flounder.

In conclusion, *amhr2* was expressed specifically in the somatic cells surrounding the germ cells during gonadal sex differentiation in both XY and XX flounder, and the loss of Amhr2 function causes male-to-female sex reversal in the flounder, along with increased *cyp19a1* and *foxl2* expressions. Moreover, fadrozole-treated *amhr2* mutants had typical testes and exhibited the down-regulation of *cyp19a1* and *foxl2* transcripts. These results strongly suggest that Amh/Amhr2 signaling causes masculinization by inhibiting estrogen synthesis during gonadal sex differentiation in the flounder.

## 4. Materials and Methods

### 4.1. Experimental Animals

The Japanese flounder (*Paralichthys olivaceus*) was used for this study. The flounder were produced artificially by mating normal males and females, and they were reared at the Nansei Field Station, Japan Fisheries Research and Education Agency. The larvae were reared at a water temperature of 16–18 °C to prevent masculinization due to high water temperatures. All of the fish were sampled after ensuring that they had been completely anesthetized by 2-phenoxyethanol (Fujifilm Wako Chemicals, Osaka, Japan). All of the experimental protocols were approved by the Institutional Animal Care and Use Committee of Nansei Field Station (2021-13).

### 4.2. Determination of Genetic Sex

The genetic sex of all of the experimental individuals was determined by genomic PCR with reference to Y-allele-specific deletion in the promoter region of *amhy* [24]. Genomic DNA was extracted from fin or gonad samples using ISOGEN*^®^* (Nippon Gene, Toyama, Japan) following the manufacturer’s instructions. A genomic PCR was carried out using AmpliTaq Gold^®^ (Applied Biosystems, Foster City, CA, USA) with the following primers: forward, 5′-GTTCAGTTCAGTTGCACAGC-3′, and reverse, 5′-AATGGGTCCAGCTTCAGAGG-3′.

### 4.3. Expression Analysis by Real-Time PCR

Total RNA was extracted from the gonads using the RNeasy Mini Kit (Qiagen, Germantown, MD, USA) following the manufacturer’s instructions. cDNA synthesis was carried out using ReverTra Ace^®^ (Toyobo, Osaka, Japan) from 100 ng of total RNA. The first strand cDNAs were diluted to 100 µL for subsequent use. A quantitative real-time PCR was performed using SYBR^®^ Green Real-time PCR Master Mix (Toyobo) and 5 μL of cDNA on an iCycler iQ (Bio-Rad, Hercules, CA, USA) with the following primers: forward for amh, 5′-TGACCCGTACCTACGAGCTG-3′, and reverse, 5′-TCGTCCACGTTCTCGCTCTC-3′; forward for amhr2, 5′-ACTGCTGGTAATGTGAGTGG-3′, and reverse, 5′-GCCCAGAATCTGCTGTAGTT-3′; forward for cyp19a1, 5′-ATCGGATCCCTGCCTGTGAC-3′, and reverse, 5′-TGGCTGATGCTCTGCTGAGG-3′; forward for foxl2, 5′-TCATCAGCAAGTTCCCCTTC-3′, and reverse, 5′-TGCCGTTGTAAGAGTTCACC-3′; forward for fshr, 5′-TCCAAACTGACAGTTCCTCG-3′, and reverse, 5′-AGAAGGCTAGGATGTTGAGG-3′; forward for elongation factor 1 alpha (ef1α), 5′-AGTTCGAGAAGGAAGCTGCC-3′, and reverse, 5′-ATCCAGAGCATCCAGCAGTG-3′; forward for α-tubulin, 5′-TGGTACGTAGGAGAGGGCAT-3′, and reverse, 5′-CCCTCTTCGTCTTCCTCGAACG-3′; forward for β-actin, 5′- ATTGCCCCACCTGAGCGTA-3′, and reverse, 5′- CATTTGCGGTGGACGATGGA-3′; forward for glyceraldehyde-3-phosphate dehydrogenase (gapdh), 5′- CGCTTCAAGGGTGAGGTCAA-3′, and reverse, 5′- TCAACCACATAGTGGGCACC-3′. The PCR conditions were as follows: initial denaturation at 95 °C for 60 s, followed by 40 cycles of 95 °C for 15 s, 60 °C for 15 s, and 72 °C for 45 s. The transcript abundance was quantified using the standard curve method with four dilution points. RefFinder [43], which integrates four specific algorithms (the comparative delta-Ct method [44], BestKeeper [45], NormFinder [46], and GeNorm [47]), was used for the assessment and screening of candidate reference genes (ef1α, α-tubulin, β-actin, and gapdh). The most stably expressed gene ef1α was used as a reference gene in this study (Supplementary Table S2).

### 4.4. Histological Analysis of amhr2 Transcripts Using In Situ Hybridization

The gonads were fixed in 4% paraformaldehyde (PFA) solution at 4 °C overnight, dehydrated in graded ethanol, embedded in paraffin, and sectioned serially at 5 μm thickness. In situ hybridization was performed using a digoxigenin (DIG)-labeled *amh* or *amhr2* RNA probe using the sections as described previously [17]. The probes for DIG-labeled RNA single strands were synthesized in vitro using DIG RNA labeling kit (Roche Diagnostics GmbH, Mannheim, Germany).

### 4.5. Generation of amhr2 Mutant Using CRISPR/Cas9 System

Synthetic crRNA and trans-activating crRNA (tracrRNA) were obtained from Fasmac Co., Ltd. (Kanagawa, Japan). The sequences of the two crRNAs for *amhr2* exons were as follows: Target 1, CAGUGACAGCAUCCAGCUGGguuuuagagcuaugcuguuuug; Target 2, UGCUUUGGGACUGCUGCUGUguuuuagagcuaugcuguuuug. These sequences were designed as described previously [25]. The two crRNAs (250 ng/µL), tracrRNA (500 ng/µL), and Cas9 protein (750 ng/µL; Nippon Gene) were mixed and injected into fertilized eggs immediately after fertilization using a microinjector (IM-9B; Narishige, Tokyo, Japan) as described previously [25]. The two crRNAs and Cas9 protein without tracrRNA were injected for the experimental control group. The artificial fertilization, microinjection, and rearing of the experimental fish were performed at Nansei Field Station, Japan Fisheries Research and Education Agency. Eggs and sperm were produced by 3-year-old females and males.

### 4.6. Analysis of Genotype in amhr2 Mutants

For genotyping, genomic DNA was extracted from the gonads using ISOGEN^®^ (Nippon Gene) following the manufacturer’s instructions. Genomic PCR was carried out using AmpliTaq Gold^®^ (Applied Biosystems) with the following primers specific for *amhr2* (forward, 5′-TCTCTTCATTCCTTTCTGTG-3′, and reverse, 5′-TAAATCTGAGCAGCGCATC-3′). The PCR conditions were as follows: preheating at 95 °C for 2 min; 35 cycles of 95 °C for 15 s, 59 °C for 30 s, and 72 °C for 2 min; then, a final extension at 72 °C for 5 min. The amplified PCR fragments were subcloned using TA PCR Cloning Kit (BioDynamics Laboratory, Tokyo, Japan) following the manufacturer’s instructions. The sequencing of subcloned amplicon PCR fragments was outsourced to a sequencing service (Fasmac Co., Ltd.).

### 4.7. Histological Observations

At 200 dah, the gonads were immediately fixed with Bouin’s solution at 4 °C overnight. The fixed gonads were dehydrated in a series of ethanol solutions and cleared using Lemosol (Fujifilm Wako Chemicals). The gonadal tissue was embedded in paraffin, sectioned serially at 4 µm thickness, stained using hematoxylin-eosin, and observed under a microscope (BZ-X700; Keyence, Osaka, Japan).

### 4.8. Experimental Treatment with Fadrozole

Fadrozole was orally administered with an artificial diet (Love Larva; Maruha Co., Yamaguchi, Japan) mixed with a 100 μg/g diet of fadrozole (Sigma-Aldrich, St. Louis, MO, USA) at 18 °C from 30 to 100 dah as described previously [7]. Phenotypic sex was determined at 200 dah by histological observation.

### 4.9. Statistical Analysis

The significance of the differences in the experimental results in the relative mRNA expressions among the various experimental groups was tested by one-way ANOVA based on normalized data, followed by Tukey’s test or Student’s *t*-test. All of the statistical analyses were performed using SPSS version 22 (IBM Corp., Armonk, NY, USA), and all of the experimental data are presented as mean ± SEM.

## Figures and Tables

**Table 1:** The types of mutations in *amhr2* knockout mutants.

				Types of Mutations		
	Genetic Sex	Phenotypic Sex (Gonad)	Number of Clones Sequenced	Wide Deletion	Short Deletion	No Mutation
wild-type	XY	♂ (Testis)	4	0	0	4
	XX	♀ (Ovary)	4	0	0	4
*amhr2* mutant	XY	♀ (Ovary)	16	15	1	0
	XY	♀ (Ovary)	16	13	3	0
	XY	♀ (Ovary)	16	15	1	0
	XX	♀ (Ovary)	8	8	0	0
	XX	♀ (Ovary)	8	6	2	0

**Table 2:** Sex ratios in wild-type and *amhr2* mutant flounder fed an fadrozole-treated diet or untreated diet (control).

Treatment	Genotype	Genetic Sex	♂ (Testis)	♀ (Ovary)
Control	wild-type	XY	9	0
Control	wild-type	XX	0	11
Control	*amhr2* mutant	XY	0	6
Control	*amhr2* mutant	XX	0	4
Fadrozole	*amhr2* mutant	XY	5	0
Fadrozole	*amhr2* mutant	XX	5	0

## Data Availability

All data are available within this article.

## Supplementary Materials

The following supporting information can be downloaded at: https://www.mdpi.com/article/10.3390/ijms24032480/s1.
