# Supplementary material for: Amh/Amhr2 Signaling Causes Masculinization by Inhibiting Estrogen Synthesis during Gonadal Sex Differentiation in Japanese Flounder (Paralichthys olivaceus)
Source: Int J Mol Sci. 2023 Jan 27;24(3):2480. doi: 10.3390/ijms24032480 (PMC9917198; doi:10.3390/ijms24032480)
Supplement: Supplementary file 1 [file ijms-24-02480-s001.zip › ijms-2162253-supplementary.pdf]

Amh/Amhr2 Signaling Causes Masculinization by Inhibiting Estrogen Synthesis during Gonadal Sex Differentiation in Japanese Flounder (*Paralichthys olivaceus*)

Supplementary Materials

Toshiya Yamaguchi[1,*] and Takeshi Kitano[2]

Supplementary Table S1. Body length and body weight in wild-type, *amhr2*-ko and fadrozole- treated *amhr2*-ko flounders at 200 dah.

	Genotypic sex	Phenotypic sex (Gonad)	Number of fish	Body length (mm)	Body weight (g)
Wild type	XY	♂ (Testis)	20	175.2 ± 8.0	23.8 ± 1.1
	XX	♀ (Ovary)	20	176.5 ± 12.6	23.9 ± 1.5
amhr2-ko	XY	♀ (Ovary)	10	178.5 ± 3.8	24.3 ± 1.3
	XX	♀ (Ovary)	10	173.6 ± 15.9	24.1 ± 1.2
amhr2-ko	XY	♂ (Testis)	5	173.6 ± 6.7	24.8 ± 1.1
Fadrozole treatment	XX	♂ (Testis)	5	176.0 ± 9.5	24.4 ± 1.1

Supplementary Table S2. Expression stability of reference genes determined by RefFinder.

Gonad type	Method	Ranking Order (Better - Good - Average)			
XY male gonad		1	2	3	4
at 60 dah	Delta-Ct	ef1α	β-actin	α-tubulin	gapdh
	BestKeeper	ef1α	α-tubulin	β-actin	gapdh
	Normfinder	ef1α	β-actin	gapdh	α-tubulin
	GeNorm	ef1α and α-tubulin		β-actin	gapdh
	total	ef1α	α-tubulin	β-actin	gapdh
XX female gonad		1	2	3	4
at 60 dah	Delta-Ct	ef1α	α-tubulin	β-actin	gapdh
	BestKeeper	ef1α	α-tubulin	gapdh	β-actin
	Normfinder	ef1α	α-tubulin	β-actin	gapdh
	GeNorm	ef1α and α-tubulin		β-actin	gapdh
	total	ef1α	α-tubulin	β-actin	gapdh
XY male testis		1	2	3	4
at 200 dah	Delta-Ct	ef1α	β-actin	gapdh	α-tubulin
	BestKeeper	ef1α	α-tubulin	β-actin	gapdh
	Normfinder	ef1α	β-actin	gapdh	α-tubulin
	GeNorm	gapdh and β-actin		ef1α	α-tubulin
	total	ef1α	β-actin	gapdh	α-tubulin
XX female ovary		1	2	3	4
at 200 dah	Delta-Ct	β-actin	ef1α	α-tubulin	gapdh
	BestKeeper	ef1α	β-actin	α-tubulin	gapdh
	Normfinder	β-actin	ef1α	α-tubulin	gapdh
	GeNorm	ef1α and α-tubulin		β-actin	gapdh
	total	ef1α	β-actin	α-tubulin	gapdh
